# Crosswinds During Spring Migration Carryover and Influence the Time Interval Between Arrival and Laying in a Neotropical Migrant

**DOI:** 10.1002/ece3.71230

**Published:** 2025-05-08

**Authors:** Michal Pavlik, Tony D. Williams, David J. Green

**Affiliations:** ^1^ Department of Biological Sciences, Centre for Wildlife Ecology Simon Fraser University Burnaby British Columbia Canada

**Keywords:** breeding phenology, carryover effects, delay between arrival and egg laying, plasma triglyceride, timing of arrival, yellow warbler

## Abstract

Events on the non‐breeding grounds and on migration can influence the timing of reproduction and the productivity of migratory songbirds. We show, using data from a 12‐year study in Revelstoke, BC, Canada, that the onset of breeding in yellow warblers ( *Setophaga petechia* ) is linked to weather conditions on migration, specifically the speed of crosswinds experienced over the western flyway during a 2‐week period from May 18 to 31. During 2015–2017, we investigated whether this carryover effect was due to crosswind effects on the timing of arrival, the reproductive state and mass of females on arrival, or a combination of other effects that delayed egg laying. In these years, female arrival dates varied with age but were independent of year, growing degree days, or crosswinds on migration. Though crosswinds experienced by females during the 14‐day period before their arrival on the breeding ground had no significant effect on their reproductive state (plasma triglyceride levels) or mass (controlling for tarsus length) on arrival, crosswinds had an effect on the time interval between arrival and egg laying. The time interval between arrival and egg laying was also shorter if females arrived with elevated plasma triglyceride levels and longer if females arrived early in the season. Carryover effects from crosswinds experienced during migration on the timing of breeding and reproductive success of yellow warblers are likely to arise due to their effect on arrival date and how rapidly birds can transition from a migratory to a reproductive physiological state. Both wind‐speed experienced on migration (14 days before arrival) and reproductive state on arrival (plasma triglyceride concentration) independently influenced the time interval between arrival and egg laying. Crosswinds that delay breeding have a significant effect on reproductive success; female yellow warblers that initiate reproduction late decrease both their chance to raise at least one nestling and the number of nestlings fledged.

## Graphical abstract

Carryover effects from crosswinds experienced during migration on the timing of breeding and reproductive success of yellow warbler ( *Setophaga petechia* ) are likely to arise due to their effect on arrival date and how rapidly birds can transition from a migratory to a reproductive physiological state. Both wind‐speed experienced on migration (14 days before arrival) and reproductive state on arrival (plasma triglyceride concentration) independently influenced the time interval between arrival and egg laying.

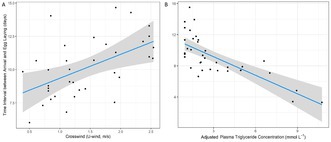

## 1 Introduction

In a seasonal environment, the timing of breeding often has consequences for reproductive success and juvenile survival (Perrins 1970; Verhulst et al. 1995). For birds, individuals that breed early often lay larger clutches (e.g., Daan et al. 1990; Crick et al. 1993), have higher nest success (e.g., Wiggins et al. 1994; Öberg et al. 2014), and are more likely to avoid depredation (e.g., de Zwaan et al. 2019) or to re‐nest after a nest fails or young fledge (e.g., Gillis et al. 2008; Hepp et al. 2021). Juveniles from early nesting attempts can also have higher survival and are more likely to recruit to the breeding population (e.g., Norris 1993; Öberg et al. 2014). However, individual decisions on when to breed are the outcome of complex trade‐offs between the costs and benefits of reproducing early for both parents and their offspring (Drent 2006) and consequently likely to vary with parental quality (e.g., age—Ratcliffe et al. 1998; condition—Descamps et al. 2011), environmental conditions on the breeding grounds (e.g., Le Vaillant et al. 2021; de Zwaan et al. 2019) and carryover effects from environmental conditions at earlier stages of the annual cycle (Harrison et al. 2010; de Zwaan et al. 2022).

Carryover effects on breeding phenology could arise due to environmental conditions before, during, and immediately after migration or the condition of individuals, which could delay the transition from a migratory to a reproductive physiology. In migratory birds, food availability on the wintering grounds can influence the timing of departure (e.g., Studds and Marra 2007, 2011; Cooper et al. 2015). Headwinds on the wintering grounds and en route can delay departure and slow migration (e.g., Grönroos et al. 2012; Rotics et al. 2018). Conditions experienced during migration can also influence arrival dates and the timing of breeding. For example, more favorable stopover conditions in North Africa advanced the arrival dates of barn swallows ( *Hirundo rustica* ) in Spain (Balbontín et al. 2009), while warm temperatures in the Mediterranean promoted earlier breeding by common redstarts ( *Phoenicurus phoenicurus* ), spotted flycatchers ( *Muscicapa striata* ) and wood warblers ( *Phylloscopus sibilatrix* ) in Europe (Finch et al. 2014). Similarly, strong winds on migration through the western United States delayed the breeding of yellow warblers ( *Setophaga petechia* ) and yellow‐breasted chats ( *Icteria virens* ) in Canada (Drake et al. 2014; Huang et al. 2017; respectively). Less is known about how events on the wintering grounds combine with the conditions experienced on migration to influence the condition of individuals when they arrive on the breeding grounds (but see de Zwaan et al. 2022; Smith and Fraser 2024). However, winter habitat use, inferred from stable carbon isotope values, can influence the arrival body condition of some songbirds (e.g., American redstart [ *Setophaga ruticilla* ; Marra et al. 1998], black‐throated blue warbler [ *Setophaga caerulescens* ; Bearhop et al. 2004], palm warbler [ *Setophaga palmarum* ; González‐Prieto and Hobson 2013]) and waterfowl (e.g., northern pintail [ *Anas acuta* ; Yerkes et al. 2008]).

Despite the high energetic costs of migration and reproduction and the potential for hormonal conflicts (Williams 2012b; Jubinville et al. 2020), females of some species can initiate the transition from a migratory to a reproductive physiology while on migration (Williams 2012a; Pavlik et al. 2021). During the non‐breeding season, bird reproductive organs are inactive and it takes several weeks to fully re‐activate them (Williams 2012b). Because of this, females begin to develop and mature their reproductive organs (ovary and oviduct) prior to or on migration (e.g., Bluhm et al. 2000; Raess and Gwinner 2005). Females of some species can also initiate vitellogenesis (a much later stage of gonadal development dependent on estrogen synthesis and secretion by the ovary) and the subsequent production of yolk‐targeted very low‐density lipoproteins (VLDLy) by the liver (Williams 2012b) during migration. For example, Crossin et al. (2010) argued that female macaroni penguins ( *Eudyptes chrysolophus* ) must initiate vitellogenesis while at sea because females lay their first egg only 7–14 days after their return to the breeding colony, whereas yolk formation is estimated to take approximately 16 days. Williams (2012a) provided more direct evidence that female surf scoters ( *Melanitta perspicillata* ) initiate vitellogenesis on migration since females had elevated blood vitellogenin levels when captured 1200 km from their breeding grounds.

Previous studies on yellow warbler suggest that climatic conditions can influence annual survival, timing of breeding, and productivity (Mazerolle et al. 2011; Drake et al. 2014). In British Columbia, stronger crosswinds during the migration period were associated with lower apparent annual survival rates and later female clutch initiation dates (Drake et al. 2014). Moreover, Pavlik et al. (2021) have recently demonstrated that some female yellow warblers (ca. 17%) can initiate vitellogenesis prior to arrival on the breeding grounds. Wind‐speed effects on the timing of breeding could therefore arise because (a) crosswinds during migration delay arrival on the breeding grounds, (b) energetic costs associated with crosswinds on migration limit the number of females that initiate vitellogenesis before arriving on the breeding grounds, or (c) crosswinds influence the condition of birds on arrival and increase the amount of time females need to build up the resources needed to produce eggs.

Here, we confirm that wind speed during migration influences the onset of reproduction and evaluate the temporal and spatial scale of wind effects on the timing of breeding. We investigate whether this carryover effect arises via effects on the reproductive physiology of females. We evaluate how environmental conditions on migration (wind speed) and on the breeding ground (growing degree days), temporal factors (arrival date), and intrinsic factors (age) influence reproductive state on arrival, estimated using plasma triglyceride concentration of females. Next, we examine how environmental, temporal, and intrinsic (age and reproductive state on arrival) factors influence the time interval between arriving on the breeding grounds and initiating reproduction. Finally, we assess the fitness consequences of the timing of breeding in yellow warblers.

## 2 Materials and Methods

### 2.1 Study System, Study Sites, and Field Methodology

The yellow warbler is a small neotropical migrant passerine with a broad breeding distribution throughout North America (Lowther et al. 1999). Our study sites were located near Revelstoke, British Columbia, Canada, and situated in the drawdown zone of the Upper Arrow Lakes Reservoir on the Columbia River (435–441 m AMSL). The three sites, approximately 24, 27 and 30 ha in size, were composed of periodically flooded riparian habitat with patches of mature black cottonwood ( *Populus trichocarpa* ) forest, willow‐dominated shrub (*Salix* spp.) and grassland.

We monitored the breeding biology of a color‐banded population of yellow warblers between 2005 and 2017. In each year, birds new to the study sites were captured in mist nets and banded with aluminum USFW bands and a unique combination of three color bands. We aged and sexed birds according to plumage criteria (Pyle 1997). We assigned each bird to one of three age categories:

- SY: second year birds—birds in their first breeding season,
- ASYL: local after second year birds—returning birds at least 2 years old that previously bred at the study sites, and
- ASYU: unknown after second year birds—new birds at least 2 years old that were new to the study population.

For all captured individuals, we measured body mass to 0.1 g using a digital scale (Insten digital pocket scale, 0.01–100 g), wing chord, tail length, and tarsus length. In all years with the exception of 2007, we documented male arrival dates and the identity of individuals in all pairs that formed and attempted to find and monitor the fate of all nests made by each female. Pairs and their nests were monitored every 1–3 days. For nests found during egg laying or after clutch completion, we estimated the date that the clutch was initiated, assuming females lay one egg per day and incubate for 11 days (mean ± SE = 11.05 ± 0.13; Martin et al. 2020). We used this information to determine the onset of reproduction (the date the first egg was laid, DFE) and the annual productivity (total number of young fledged from all nesting attempts) for 16–35 females per year (*n* = 305 female years in total). More details about the three study sites and the breeding biology of this population are provided by Quinlan and Green (2012) and Hepp et al. (2021).

In 2015–2017, we increased our monitoring effort and also documented female arrival dates (date of arrival, DOA). In these 3 years, study sites were visited every 1–2 days (typically, two sites were visited each day for 2–5 h per site) from the beginning of May to late July. We attempted to capture all females on the day they were first observed or as soon as possible thereafter. Females (*n* = 83) were captured using mist nets combined with call and/or song playback. We assigned each female an arrival date, assuming it arrived on the day it was first observed if we had visited the site the previous day or the day before, and it was first observed if there was a 2‐day interval between site visits. We then used these arrival dates to calculate the number of days between arrival and capture (days after arrival, DAA) and the time interval between arrival and when females laid their first egg of the season (egg after arrival, EAA).

### 2.2 Physiological State of Females on Arrival

Physiological state of females when they arrived on the breeding ground was assessed by measuring their plasma triglyceride levels. While migration requires low (< 3 mmol L^−1^) levels of triglycerides (generic very low‐density lipoproteins or generic VLDLs), the transition to a reproductive physiology is associated with a dramatic increase in circulating triglyceride levels (> 10 mmol L^−1^) as estrogens trigger the production of vitellogenin (VTG) and yolk‐targeted very low‐density lipoproteins (VLDLy) by the liver (Vanderkist et al. 2000; Challenger et al. 2001; Williams 2012b). For yellow warbler, baseline plasma VLDL levels are ca. 1.6 mmol L^−1^, but increase to ca. 9.5 mmol L^−1^ three days before egg laying, peaking at ca. 14.2 mmol L^−1^ (Pavlik et al. 2021). This shift in lipid metabolism from producing low levels of generic VLDL that fuel migration to high levels of VLDLy necessary for vitellogenesis and egg formation allows plasma triglyceride levels to be used as an index of egg‐yolk precursor production and vitellogenesis (Williams 2012b; Crossin and Williams 2021).

Blood samples (< 75 μL) were collected in heparinized 50‐μL microcapillary tubes after puncturing the brachial vein with a 26‐gauge sterile needle. All birds were sampled within 20 min of capture, and the blood samples were stored in a cooler with ice for no more than 4 h before separating the plasma from the red blood cells by centrifuging the microcapillary tubes for 10 min at 12,000 rpm. Plasma samples were then stored in a freezer at −20°C until they were analyzed.

Plasma triglyceride concentration was determined following an established protocol described in detail by Williams et al. (2007). This protocol corrects for the presence of free glycerol by subtracting the free glycerol concentration from the total triglyceride concentration. We determined the concentration of free glycerol and total triglyceride using a Sigma‐Aldrich free glycerol reagent kit—F6428 and triglyceride reagent kit—T2449, respectively. To determine free glycerol concentration, 5 μL of plasma was pipetted into wells of a 96‐well microplate (400 μL flat‐bottom) and 240 μL of the free glycerol reagent was added to each well. Microplates were then shaken for 30 s and incubated for 10 min at 37°C. Each plate was then read in the absorbance plate reader (Bio‐Tek PowerWave 340) to determine optical density measurements of samples from which free glycerol concentration was determined using a standard curve. The standard curve for each plate was estimated from values obtained from a serial dilution of a 2.54‐mmol glycerol standard (Sigma‐Aldrich G7793). To establish the total triglyceride concentration, 60 μL of the triglyceride reagent was added to each well, followed by an additional 30‐s shake, 10‐min incubation at 37°C, and the plates were re‐read in the plate reader.

A hen plasma pool was included in each assay to assess intra‐ and inter‐year assay variation. For free glycerol assays, the intra‐assay coefficient of variation was 5.6% and the inter‐assay coefficient of variation was 8.4%. For total triglyceride assays, the intra‐assay coefficient of variation was 2.8% and the interannual coefficient of variation was 3.8%. All assays were run in duplicate and the mean plasma triglyceride concentration was used in analyses.

### 2.3 Wind Speed on the Migration Flyway

The wind conditions experienced during spring migration were quantified using modeled wind‐speed data extracted from the National Center of Environmental Prediction (NCEP) Reanalysis 1 data archives at the NOAA‐CIRES Climate Diagnostics Center at Boulder, Colorado, USA (Kalnay et al. 1996) using the RNCEP package for R statistical software (Kemp et al. 2012). These data have a spatial resolution of 2.5° latitude and longitude and a temporal resolution of 6 h. Because songbirds migrate at night and mostly at elevations from close to the surface up to 2100 m (Alerstam et al. 2011; Bruderer et al. 2018) depending on terrain and environmental conditions, we used average nighttime (1800, 0000, and 0600) westerly (U‐wind) and southerly (V‐wind) components at the 850 mb (~1500 m AMSL) and 925 mb (~700 m AMSL) levels. For the crosswind (U‐wind) absolute values of the wind vector were used; however, for the tailwind (V‐wind), original wind values were used, reflecting a difference between headwind (negative values) and tailwind (positive values).

**FIGURE 1:**
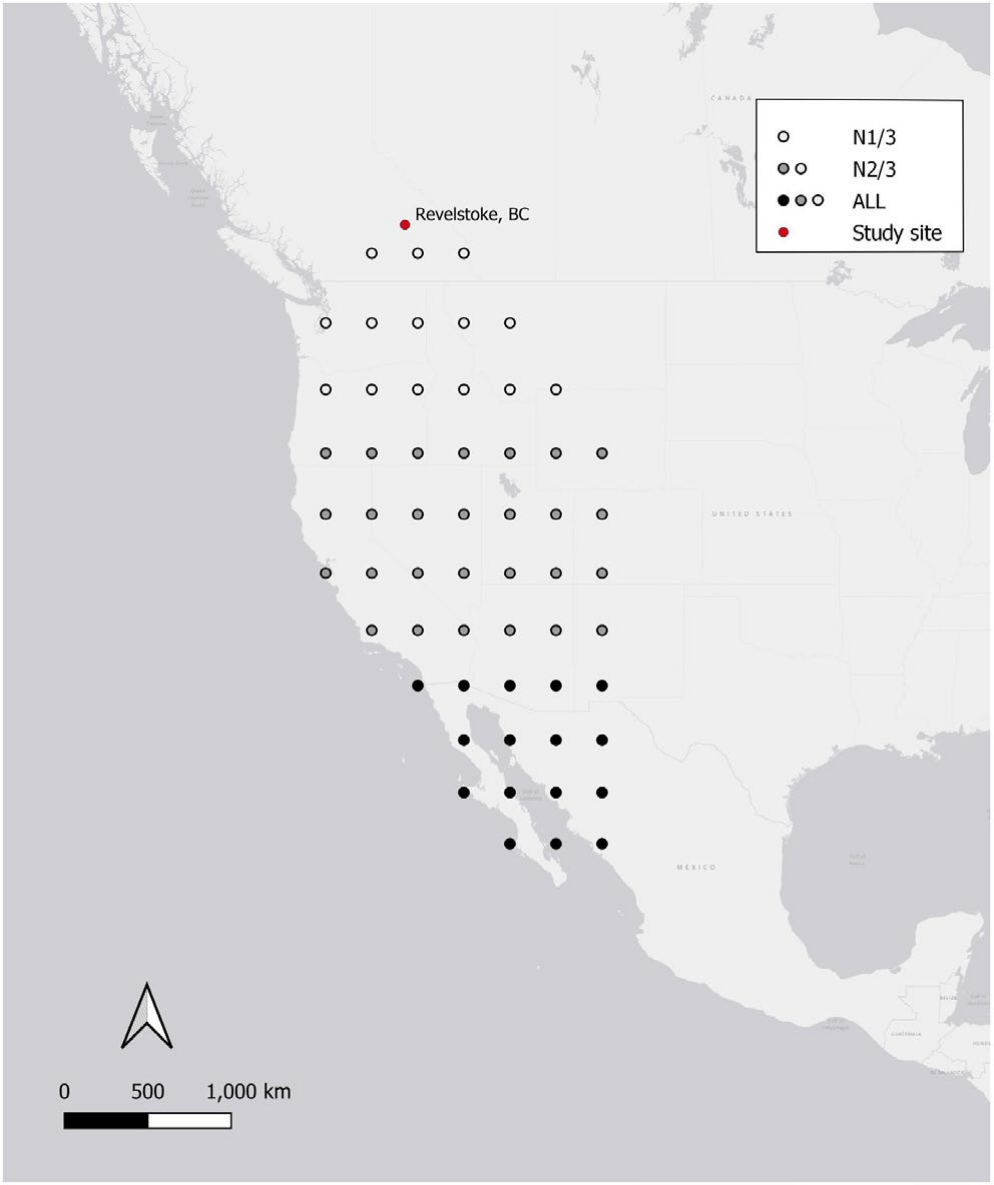
The location of the study area in Revelstoke, BC and the points used to calculate the wind speed values during migration for the three geographical areas (N1/3 = the northern 1/3 of the flyway, N2/3 = the northern 2/3 of the flyway, and ALL = the entire flyway).

We defined the western flyway for our population as the overland region west of the easternmost portion of the continental divide (107° W), beginning at the northern extent of the yellow warbler wintering range (25° N) and ending at the latitude of our study site (50° N). We calculated the average annual U‐wind and V‐wind components for three geographical areas:

- ALL: the entire flyway area (25° N—50° N),
- N2/3: the northern 2/3 of the flyway (35° N—50° N), and
- N1/3: the northern 1/3 of the flyway (45° N—50° N; Figure 1).

Each of these areas had four temporal windows: (1) 2 months (April–May), (2) 1 month (May), (3) 14 days (May 18–31), and (4) 7 days (May 25–31). We chose the geographical area and temporal window based on the best model in the candidate set examining spatiotemporal effects of wind conditions on lay dates in this population (see below and Section 3). We also calculated the likely crosswinds experienced by each individual on their northern migration by averaging the U‐wind components over the entire flyway over the 14 days prior to their arrival on the breeding grounds.

### 2.4 Environmental Conditions on the Breeding Ground

For each year, we used growing degree days (GDD) as a measure of thermal accumulation over time that reliably predicts plant phenology on the breeding ground (e.g., bud break in trees; Hakkinen et al. 1998; Linkosalo 1999), which in turn supports the arthropod prey abundance of yellow warblers (e.g., Hodgson et al. 2011; Cayton et al. 2015). GDD was calculated as a cumulative sum of mean daily temperatures exceeding 5°C (i.e., threshold as in Hodgson et al. 2011) at the start of the breeding season (set as June 1) and for the day when individual females arrived on the breeding grounds in 2015 to 2017:

$$\mathrm{GDD}=\mathrm{Sum}\;\left(\frac{\left(\text{Tmax}+\text{Tmin}\right)}{2}-5\right)$$

for each day from Jan 1 to May 31 or Jan 1 to Arrival Date.

Daily maximum and minimum temperatures for Revelstoke (Revelstoke A station, 50°58′ N, 118°11′ W; WMO id = 71,685) were downloaded from the Environment and Climate Change Canada website (https://climate.weather.gc.ca/historical_data/search_historic_data_e.html?Month=12&Day=4&Year=2022&timeframe=2&StartYear=1840&EndYear=2022).

### 2.5 Statistical Analyses

We conducted our analyses using two datasets. The first included data on the onset of reproduction (the date of the first egg) and the annual productivity of female yellow warblers (*n* = 226) from 2005 to 2017 (*n* = 305 female years). The second included data from the intensive monitoring of female yellow warblers from their arrival to the onset of reproduction in 2015 to 2017. We used the first dataset to (i) determine the spatiotemporal window that best explained wind speed on migration effects on the onset of reproduction and (ii) evaluate how the timing of breeding (date of first egg) influenced annual productivity. We used the second dataset to examine variation in (i) female arrival date, (ii) female reproductive state (plasma triglyceride level), (iii) mass on arrival, and (iv) the time interval from arrival to egg laying.

We created two candidate model sets using the long‐term dataset. The first candidate model set (*n* = 13 models) examined spatiotemporal effects of wind‐speed on the date of the first egg. The linear mixed effect models all included the two wind components (crosswinds, U‐wind, tailwinds, and V‐wind) and age class (SY, ASYU, and ASYL) as fixed effects and individual ID and Year as random terms. Wind components (U‐wind_year_ and V‐wind_year_) were standardized to a mean of zero and a standard deviation of one. Candidate models differed in the geographic area (northern 1/3, northern 2/3, and ALL of the flyway) and temporal window used to calculate the average crosswind and tailwind (2 months, April to May; 1 month, May; 14 days, May 18–31; and 7 days, May 25–31). We did not include quadratic wind‐speed terms as preliminary analyses found no evidence that quadratic terms improved the models. The second candidate model set (*n* = 8 models) examined the fitness consequences associated with advancing the onset of reproduction. We examined age class, the date of first egg, and year effects on variation in annual productivity (the total number of fledged nestlings) using “hurdle” models (Zeileis et al. 2008) where a binomial distribution was used to model failure/success and a Poisson distribution was used to model the number fledged. The candidate model set included models with all combinations of age class, date of first egg (standardized to a mean of zero and a standard deviation of one), year as main effects, and a null model. We did not include quadratic date terms as preliminary analyses found no evidence that quadratic terms improved the models.

We created four candidate model sets using the intensive monitoring dataset. The first candidate model set (*n* = 10 models) examined annual variation in the arrival date of females (*n* = 65) in the 3 years with more extensive monitoring (*n* = 81 female years). The linear mixed effect models in this candidate set included combinations of age class (SY, ASYU, and ASYL), year, average crosswinds across the entire flyway from May 18 to 31 (U‐wind_year_), and cumulative growing degree days at the start of the breeding season (GDD_year_). We used average crosswinds on the entire flyway from May 18 to 31 as this best‐described wind effects on the onset of reproduction (see above and Section 3). We excluded average tailwinds as this variable had no effect on the onset of reproduction (see Section 3). Average crosswinds and cumulative growing degree days were standardized to a mean of zero and a standard deviation of one. All models included individual ID as a random term. The candidate model set included all univariate models, bivariate models with age, and a null model. The second and third candidate model sets (*n* = 15 models) examined variation in the plasma triglyceride levels or mass of female yellow warblers (*n* = 37 and *n* = 35, respectively) captured on or within 2 days of arrival; *n* = 42 female years for triglyceride and *n* = 40 female years for mass. Triglyceride values were log‐transformed to conform to the assumptions of normality and heterogeneity. Mass values were residuals from a mass‐tarsus regression. The linear mixed effect models in each candidate set included combinations of age class, year, arrival date, average crosswinds on the entire flyway in the 2 weeks prior to each female's arrival (U‐wind_ind_), and cumulative growing degree days at the time individual females arrived (GDD_ind_). Arrival date, average crosswinds, and cumulative growing degrees were all standardized to a mean of zero and a standard deviation of one. We used average crosswinds over the entire flyway in the 2 weeks prior to arrival since this spatiotemporal scale best‐described wind effects on the onset of reproduction (see above). We did not include models with both arrival date and cumulative growing degree days in the candidate set since these variables were strongly correlated. Models examining variation in the plasma triglyceride levels included a term to control for the timing of capture (0–1 or 2 days after arrival). All models in both candidate sets included individual ID as a random term. The fourth candidate set (*n* = 30 models) examined variation in the time interval between arrival date and egg laying (the date of first egg; *n* = 31 females, *n* = 35 female years). The general linear models in this candidate set included combinations of age class, year, arrival date, average crosswinds on the entire flyway in the 2 weeks prior to each female's arrival (U‐wind_ind_), cumulative growing degree days at the time individual females arrived (GDD_ind_), adjusted plasma triglyceride levels, and adjusted mass. Adjusted plasma triglyceride levels were controlled for the timing of capture while adjusted mass was controlled for the tarsus length. Arrival date, average crosswinds, cumulative growing degrees, adjusted plasma triglyceride levels, and adjusted mass were all standardized to a mean of zero and a standard deviation of one. We did not include models with arrival date and cumulative growing degree days since these variables were strongly correlated. We were unable to include individual ID as a random term in these models as linear mixed‐effect models failed to converge.

**FIGURE 2:**
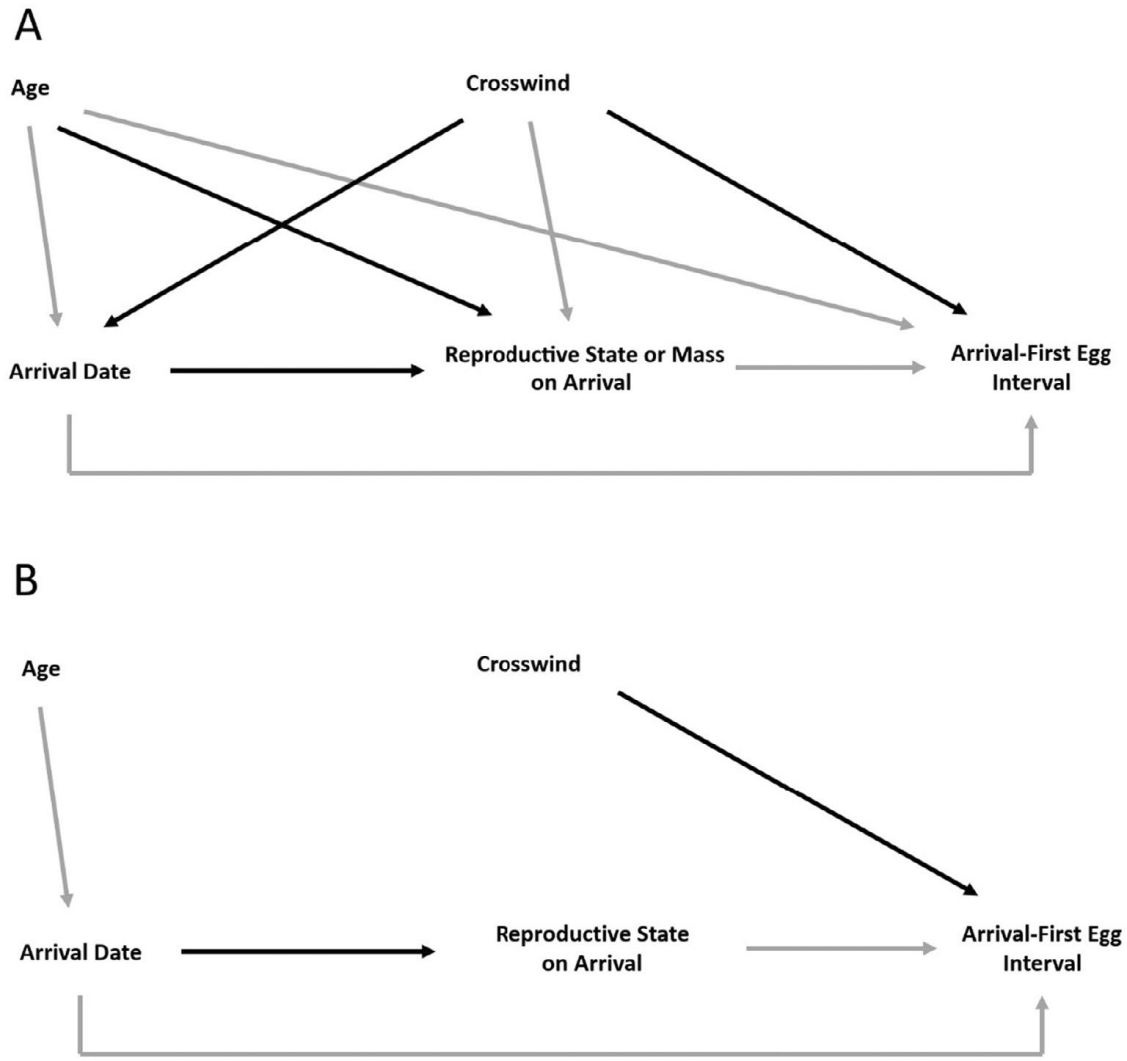
Predicted effects of age and crosswind on arrival date, reproductive state on arrival, and arrival‐egg laying interval (A). Relationships confirmed by our analyses (B). Solid black lines represent a positive relationship, and solid gray lines represent a negative relationship.

All analyses were conducted using the R statistical software (R Core Team 2022). Mixed effects models were fitted using “lmer” function from “lme4” package (Bates et al. 2015), and hurdle models were run using “hurdle” function from “pscl” package (Zeileis et al. 2008). All parameters of the models were estimated using the maximum likelihood (ML) method. Models in the candidate set were ranked using Akaike's Information Criterion corrected for small sample sizes (Burnham and Anderson 2002). Relationships in the top models were visualized using the package “visreg” within the R statistical framework (Breheny and Burchett 2017). A simplified path diagram summarizing predicted relationships from all analyses is provided in Figure 2.

## 3 Results

**FIGURE 3:**
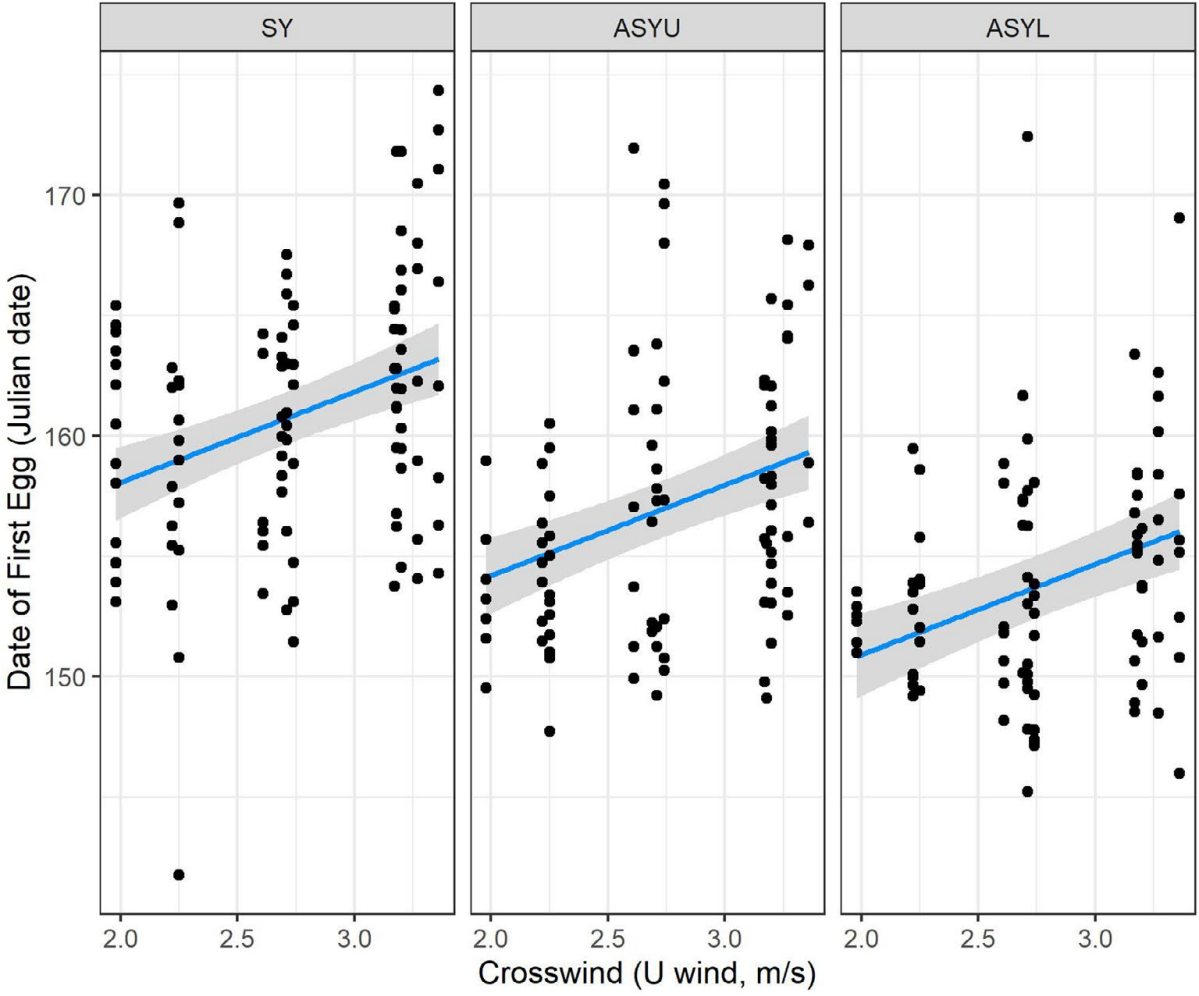
Relationship between crosswind (U‐wind) speed on the migration flyway during the 14‐day period May 18–31 and the date of the first egg for yellow warblers breeding in Revelstoke, British Columbia, from 2005 to 2017. SY females are second‐year females breeding for the first time, ASYU are after second‐year females of at least 2 years of age that are new to the study area, and ASYL are local after second‐year females that are returning to the study area. We present the model predictions (line), 95% confidence interval (shading), and partial residuals (points) from the top model in the candidate set that included age class, standardized average tailwind (V‐wind), and crosswind (U‐wind) speed as fixed effects and individual ID and Year as random terms.

The top model in the candidate set examining spatiotemporal effects on the onset of reproduction suggested that the variation in the date of the first egg laid by females between 2005 and 2017 was best described by the model with wind speeds over the entire flyway during the 14‐day period (May 18–31). This model received substantially more support than models with a larger time window or the null model with no migration wind effects (Table 1). The date of the first egg tended to be later as crosswinds increased (standardized crosswind effect [± SE] = 1.70 ± 0.35 [95% CI = 1.02–2.38]). An increase in average crosswind speed of 1 m/s delayed the date of the first egg by 3.76 days (95% CI = 2.26–5.27) (Figure 3). The date of the first egg was not impacted by tailwinds (V‐wind; standardized effect [± SE] = −0.19 ± 0.34 [95% CI = −0.87 to 0.49]; Figure S1). The date of the first egg also varied with age class (Figure S2). Older returning local females laid their first egg before older females that had not been banded and were likely new to the study population and young females breeding for the first time (ASYU female by 3.29 days [95% CI = 1.59–5.00] and SY female by 7.16 days [95% CI = 5.49–8.83]).

**TABLE 1:** Comparison of support for models in the candidate set examining spatiotemporal effects of crosswind (U‐wind) and tailwind (V‐wind) speed on the date of the first egg of female yellow warblers breeding in Revelstoke, British Columbia, from 2005 to 2017. Models vary in the geographic area and temporal time window used to calculate standardized, average U and V‐wind components. All models also include age class as a fixed effect and individual ID and Year as random terms. Models are ranked according to the difference from the best model based on Akaike's Information Criterion corrected for small sample size (AICc). *K* is the number of model parameters, and *w*_*i*_ is the Akaike weight. N1/3 = the northern 1/3 of the flyway, N2/3 = the northern 2/3 of the flyway, and ALL = the entire flyway.

Model	*K*	AICc	∆AICc	*w*_*i*_
ALL − 14 days + Age	8	1971.58	0	0.69
ALL − 1 month + Age	8	1974.10	2.52	0.20
N2/3 − 14 days + Age	8	1976.42	4.84	0.06
ALL − 2 months + Age	8	1977.79	6.21	0.03
N2/3 − 7 days + Age	8	1981.35	9.77	0.01
N2/3 − 2 months + Age	8	1981.46	9.88	0
N1/3 − 2 months + Age	8	1982.12	10.54	0
N2/3 − 1 month + Age	8	1983.10	11.52	0
ALL − 7 days + Age	8	1983.78	12.20	0
Null (Age only)	6	1985.98	14.40	0
N1/3 − 7 days + Age	8	1986.25	14.67	0
N1/3 − 14 days + Age	8	1988.24	16.66	0
N1/3 − 1 month + Age	8	1988.46	16.88	0

The top model in the candidate set examining variation in female arrival dates in the 3 years from 2015 to 2017 included only the age class term. The top model received more than 2.5 times the support of models with year, cumulative growing degree days at the start of the breeding season, or average crosswind speed over the entire flyway from May 18 to 31 (Table 2). Older females, whether they had been banded previously or not, arrived before young females (ASYU females by 5.91 days [95% CI = 3.35–8.46] and ASYL females by 6.72 days [95% CI = 4.10–9.31]; Figure S3). For these 3 years (2015, 2016, and 2017), there was some variation in growing degree days at the start of the breeding season but little variation in average crosswind speed.

**TABLE 2:** Comparison of support for models in the candidate set examining variation in the arrival dates of female yellow warblers breeding in Revelstoke, British Columbia, from 2015 to 2017. All models include individual ID as a random term. Models are ranked according to the difference from the best model based on Akaike's Information Criterion corrected for small sample size (AICc). *K* is the number of model parameters, and *w*_*i*_ is the Akaike weight. Age = age class, GDD_year_ = cumulative growing degree days at the start of breeding season, U‐wind_year_ = average crosswind across the entire flyway from May 18 to 31.

Model	*K*	AICc	∆AICc	*w*_*i*_
Age	5	497.69	0	0.52
Age + GDD_year_	6	499.66	1.97	0.19
Age + U‐wind_year_	6	499.86	2.17	0.17
Age + Year	7	501.82	4.13	0.07
Age*GDD_year_	8	503.24	5.55	0.03
Age*U‐wind_year_	8	504.25	6.56	0.02
Age*Year	11	510.55	12.86	0
Null	3	518.91	21.22	0
GDD_year_	4	520.31	22.62	0
U‐wind_year_	4	520.90	23.21	0

**FIGURE 4:**
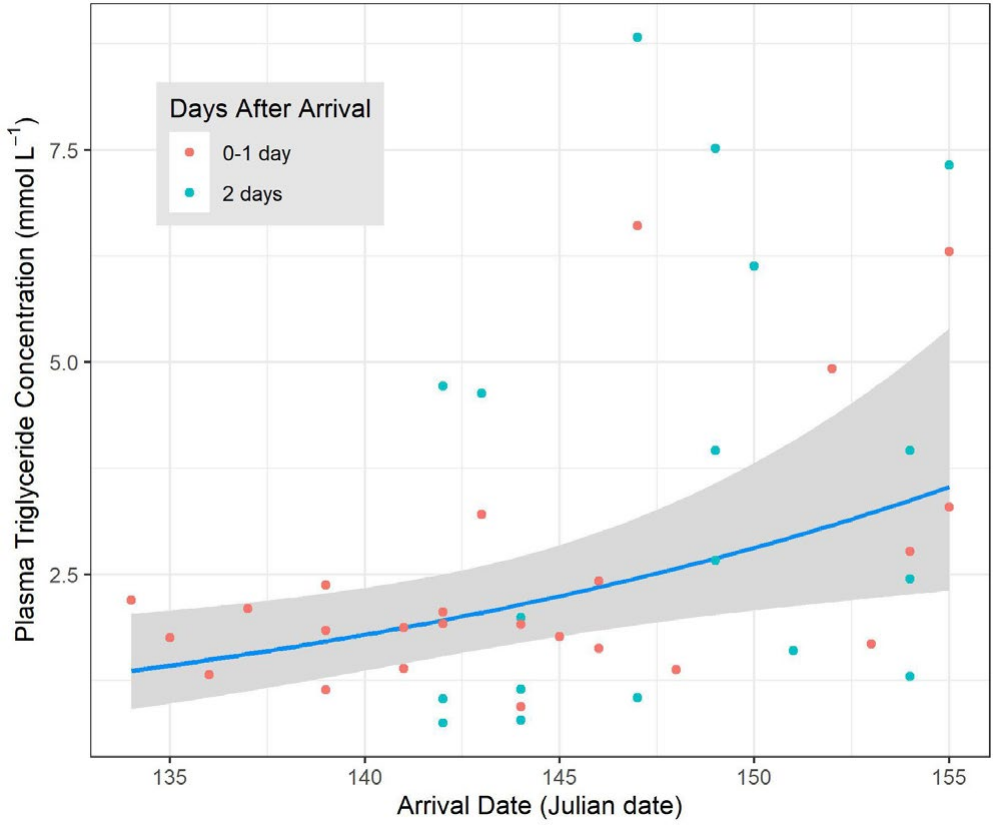
Relationship between the arrival date and the plasma triglyceride concentration of female yellow warblers captured within 2 days of arrival on the breeding grounds in Revelstoke, British Columbia (*n* = 42). We present the model predictions (line), 95% confidence interval (shading), and partial residuals (points) from the top model in the candidate set that included the standardized arrival date and the timing of capture (days after arrival, DAA) as fixed effects and individual ID as a random term.

The top model in the candidate set examining variation in the plasma triglyceride concentrations of female yellow warblers captured within 2 days of arrival included the date of arrival and timing of capture (days after arrival, DAA). This model received two times the support of the model that also included the crosswind (U‐wind) term (Table 3). Females that arrived later had higher plasma triglyceride levels (log‐transformed) than those arriving earlier in the season (standardized arrival date effect [± SE] = 0.27 ± 0.10 [95% CI = 0.07–0.46]; Figure 4). Females captured 2 days after arrival also tended to have higher plasma triglyceride levels (log‐transformed) than those captured earlier (days after arrival effect [± SE] = 0.32 ± 0.19 [95% CI = −0.06—0.71]). The model that included the crosswind term in addition to the arrival date and day of capture terms, received some support. However, the estimated effect of crosswind was slight (standardized effect [± SE] = −0.13 ± 0.11 [95% CI = −0.36 0.09], Figure S4). Models that included other terms received little support (Table 3).

**TABLE 3:** Comparison of support for models in the candidate set examining variation in the plasma triglyceride concentration of female yellow warblers captured within 2 days of arrival on the breeding grounds in Revelstoke, British Columbia, from 2015 to 2017. All models control for the time of capture (days after arrival, DAA) and include individual ID as a random term. Models are ranked according to the difference from the best model based on Akaike's Information Criterion corrected for small sample size (AICc). *K* is the number of model parameters, and *w*_*i*_ is the Akaike weight. DOA = arrival date, U‐wind_ind_ = average crosswind on the entire flyway in the 2 weeks prior to each female's arrival, GDD_ind_ = cumulative growing degree days at the time of individual female arrival, Age = age class.

Model	*K*	AICc	∆AICc	*w*_*i*_
DOA (+DAA)	5	86.14	0	0.50
DOA + U‐wind_ind_ (+DAA)	6	87.53	1.39	0.25
DOA + Year (+DAA)	7	90.24	4.09	0.07
Null (DAA)	4	90.46	4.32	0.06
DOA + Age (+DAA)	7	91.76	5.61	0.03
GDD_ind_ (+DAA)	5	92.29	6.15	0.02
U‐wind_ind_ (+DAA)	5	92.87	6.73	0.02
DOA + Age + U‐wind_ind_ (+DAA)	8	93.48	7.34	0.01
Year (+DAA)	6	94.00	7.86	0.01
Age (+DAA)	6	94.44	8.30	0.01
Year + U‐wind_ind_ (+DAA)	7	94.68	8.53	0.01
GDD_ind_ + U‐wind_ind_ (+DAA)	6	95.02	8.88	0.01
Age + GDD_ind_ (+DAA)	7	97.04	10.89	0
Age + U‐wind_ind_ (+DAA)	7	97.33	11.18	0
Age + Year (+DAA)	8	98.34	12.19	0

The top model in the candidate set examining the mass (residuals from a mass‐tarsus regression) of female yellow warblers captured within 2 days of arrival only included the cumulative growing degree days on arrival (GDD_ind_) term. This model received more than two times the support of the model that only included the crosswind term (Table 4). Female mass, controlling for tarsus length, on arrival was higher when the cumulative growing degree days on arrival were higher (standardized GDD_ind_ effect [± SE] = 0.17 ± 0.07 [95% CI = 0.02–0.32]; Figure S5).

**TABLE 4:** Comparison of support for models in the candidate set examining variation in the mass of female yellow warblers captured within 2 days of arrival on the breeding grounds in Revelstoke, British Columbia, from 2015 to 2017. Mass estimates are residuals from a mass‐tarsus regression. All models include individual ID as a random term. Models are ranked according to the difference from the best model based on Akaike's Information Criterion corrected for small sample size (AICc). *K* is the number of model parameters, and *w*_*i*_ is the Akaike weight. DOA = arrival date, U‐wind_ind_ = average crosswind on the entire flyway in the 2 weeks prior to each female's arrival, GDD_ind_ = cumulative growing degree days at the time of individual female arrival, Age = age class.

Model	*K*	AICc	∆AICc	*w*_*i*_
GDD_ind_	4	62.70	0	0.36
U‐wind_ind_	4	65.02	2.31	0.11
GDD_ind_ + U‐wind_ind_	5	65.04	2.33	0.11
Null	3	65.27	2.57	0.10
DOA	4	65.74	3.03	0.08
Year + U‐wind_ind_	6	66.60	3.89	0.05
Year	5	67.13	4.42	0.04
Age + GDD_ind_	6	67.17	4.47	0.04
DOA + U‐wind_ind_	5	67.36	4.66	0.04
DOA + Year	6	67.67	4.97	0.03
Age + U‐wind_ind_	6	69.80	7.10	0.01
DOA + Age	6	69.88	7.17	0.01
Age	5	69.97	7.26	0.01
DOA + Age + U‐wind_ind_	7	71.95	9.24	0
Age + Year	7	72.51	9.81	0

**FIGURE 5:**
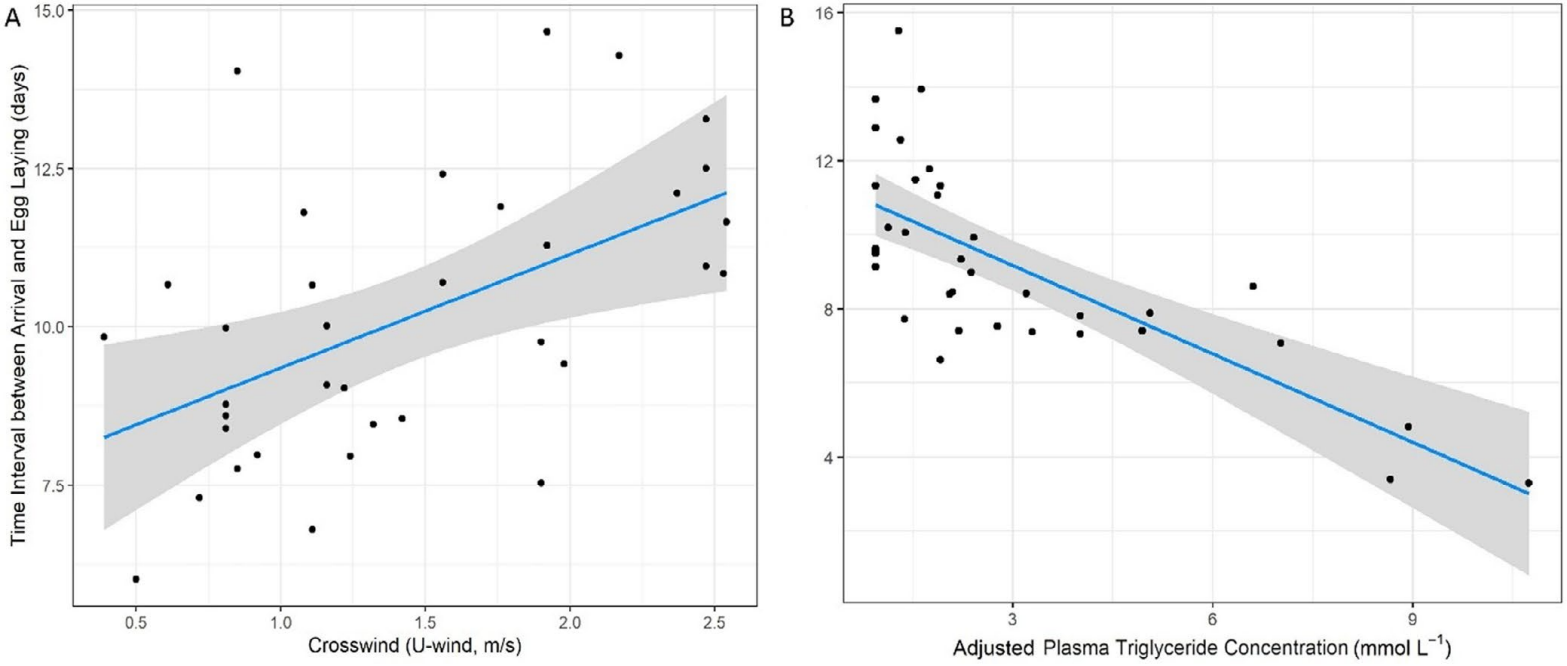
Relationship between (A) the crosswind (U‐wind) speed experienced by female yellow warblers and (B) the plasma triglyceride concentration of female yellow warblers sampled within 2 days of arrival on the breeding grounds (*n* = 36) and the time interval between arrival and egg laying. We present the model predictions (line), 95% confidence interval (shading), and partial residuals (points) from the top model in the candidate set that included the standardized crosswind (U‐wind_ind_), standardized adjusted plasma triglyceride concentration (Trig_adj_) and standardized arrival date terms.

The top model in the candidate set describing variation in the time interval between arrival and egg laying in female yellow warblers included the crosswind, adjusted triglyceride, and arrival date terms. This model received almost three times the support of the next best model, which did not include the crosswind term (Table 5). The top model suggested that crosswinds experienced on migration during the 14‐day period before arrival on the breeding grounds increased the time interval between arrival and egg laying (standardized crosswind effect [± SE] = 1.18 ± 0.40 [95% CI = 0.37–1.99]) with a 1 m/s increase in average crosswind speed estimated to delay laying by 1.79 days (95% CI = 0.58–3.00; Figure 5A). This model also suggested that females arriving with higher plasma triglyceride levels or arriving later were able to reduce the arrival‐first egg interval (standardized triglyceride effect [± SE] = −2.03 ± 0.34 [−2.72 to −1.34] and standardized arrival date effect = −1.16 ± 0.40 [−1.97 to −0.34]). A 1 mmol/L increase in plasma triglyceride concentration was estimated to advance egg laying by 0.80 days (95% CI = 0.53–1.06; Figure 5B), and a 1‐day delay in arrival advanced laying by 0.17 day (95% CI = 0.05–0.29).

**TABLE 5:** Comparison of support for models exaimining the interval between arrival and egg laying in female yellow warblers captured within 2 days of arrival on breeding grounds in Revelstoke, British Columbia. Models are ranked according to the difference from the best model based on Akaike's Information Criterion corrected for small sample size (AICc). *K* is the number of model parameters, and *w*_*i*_ is the Akaike weight. DOA = arrival date, U‐wind_ind_ = average crosswind on the entire flyway in the 2 weeks prior to each female's arrival, GDD_ind_ = cumulative growing degree days at the time of individual female arrival, Trig_adj_ = adjusted triglyceride controlled for the timing of capture, Mass_adj_ = mass controlled for tarsus length, Age = age class.

Model	*K*	AICc	∆AICc	*w*_*i*_
U‐wind_ind_ + Trig_adj_ + DOA	5	153.14	0	0.60
Year + Trig_adj_	5	155.08	1.94	0.23
Trig_adj_	3	158.62	5.47	0.04
U‐wind_ind_ + Trig_adj_	4	158.72	5.57	0.04
DOA + Trig_adj_	4	159.17	6.03	0.03
Year + Mass_adj_	5	159.71	6.56	0.02
Year + Trig_adj_ + Age	7	160.62	7.47	0.01
GDD_ind_ + Trig_adj_	4	160.85	7.71	0.01
U‐wind_ind_ + GDD_ind_ + Trig_adj_	5	161.41	8.27	0.01
Trig_adj_ + Age	5	163.90	10.76	0
U‐wind_ind_ + Mass_adj_ + DOA	5	168.93	15.79	0
U‐wind_ind_ + Mass_adj_	4	170.16	17.02	0
U‐wind_ind_ + Mass_adj_ + GDD_ind_	5	171.00	17.86	0
Mass_adj_	3	172.19	19.05	0
Year + DOA	5	173.66	20.51	0
DOA + Mass_adj_	4	174.26	21.12	0
Age + Mass_adj_	5	176.16	23.02	0
Year	4	177.30	24.15	0
U‐wind_ind_ + DOA	4	177.33	24.19	0
DOA	3	178.35	25.21	0
U‐wind_ind_ + Year	5	178.72	25.58	0
Null	2	179.61	26.46	0
U‐wind_ind_	3	181.81	28.67	0
GDD_ind_	3	181.94	28.80	0
Age + Year	6	182.53	29.38	0
Age + DOA	5	183.23	30.09	0
Age	4	183.98	30.84	0
U‐wind_ind_ + GDD_ind_	4	184.37	31.23	0
U‐wind_ind_ + Age	5	186.42	33.28	0
GDD_ind_ + Age	5	186.61	33.46	0

**FIGURE 6:**
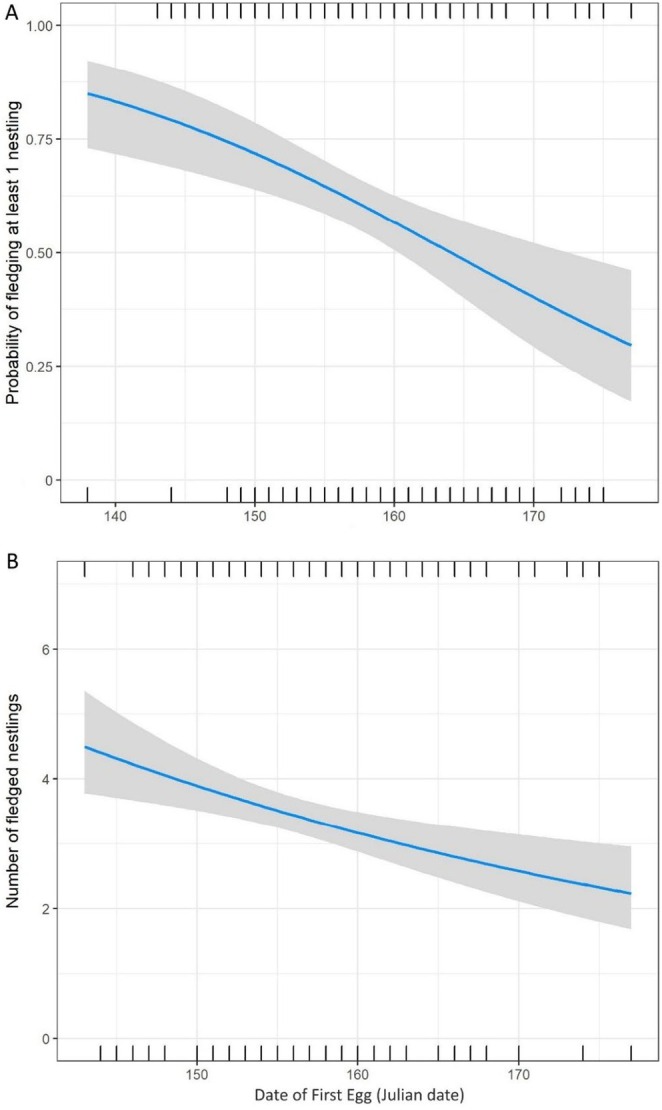
Relationship between the timing of breeding (date of first egg) and (A) the probability of fledging at least one nestling and (B) the number of fledged nestlings (if successful) for female yellow warblers breeding in Revelstoke, British Columbia, from 2005 to 2017. We present the model predictions (line) and 95% confidence interval (shading) from the top model that included the standardized date of the first egg term.

The top hurdle model in the candidate set examining variation in the annual productivity of female yellow warblers and contained only the date of the first egg term. This model received more than tthree times the support of the next best model, which also included the age class term (Table 6). Laying early increased both the probability of fledging at least one nestling (standardized effect [± SE] = −0.07 ± 0.02 [−0.10 to −0.03] Figure 6A) and the number of young fledged if females were successful (standardized effect [± SE] = −0.02 ± 0.01 [−0.04 to −0.01] Figure 6B).

**TABLE 6:** Comparison of support for hurdle models in the candidate set examining variation in the annual productivity of yellow warblers breeding in Revelstoke, British Columbia, from 2005 to 2018. Models are ranked according to the difference from the best model based on Akaike's Information Criterion corrected for small sample size (AICc). *K* is the number of model parameters, and *w*_*i*_ is the Akaike weight. DFE = date of first egg. Age = Age class.

Model	*K*	AICc	∆AICc	*w*_*i*_
DFE	4	1023.00	0	0.79
DFE + Age	8	1025.71	2.71	0.21
Age	6	1036.44	13.44	0
Null	2	1046.01	23.01	0
DFE + Year	26	1058.19	35.19	0
DFE + Age + Year	30	1063.75	40.75	0
Age + Year	28	1074.93	51.93	0
Year	24	1081.95	58.95	0

## 4 Discussion

In many migratory species, females gain a fitness benefit from initiating reproduction early in the breeding season (Perrins 1970; Wiggins et al. 1994; Newton 1998; Öberg et al. 2014). Female yellow warblers are no exception; females that initiate reproduction early increase both their chance to raise at least 1 nestling and the number of nestlings fledged. In this study, we confirm that the timing of breeding in yellow warblers is linked to weather conditions during migration, specifically the crosswind speed experienced during northward migration. We show that carryover effects of crosswind speed on the onset of reproduction could be explained, in part, by their influence on the time interval between arrival on the breeding grounds and when females lay their first egg.

Potential carryover effects of wind during the migration period depend both on the timing and location of wind events and the amount of time migrating birds are exposed to them. Previous studies have investigated these carryover effects at the population level using interannual variation in the date of the first egg and average wind‐speed values during the months migration is thought to occur across the entire flyway (Drake et al. 2014; Huang et al. 2017). In this study, we show that at the population level, a shorter time period (2 weeks) performed better than longer periods (1 or 2 months). At the individual level, crosswinds faced in the 14 days before an individual's arrival on the breeding grounds was correlated with the interval between arrival and egg laying but not with the triglyceride concentration of newly arrived females. These results are not entirely unexpected, as multiple tracking studies show that neotropical migrant songbirds can travel at the rate of 250–350 km/day during their spring migration (Stutchbury et al. 2009; Heckscher et al. 2011; Gallo et al. 2013) and can complete their spring migration within 14 days. For example, during spring migration, wood thrushes ( *Hylocichla mustelina* ) were documented to complete their ~3700 km migration within 13–15 days (Stutchbury et al. 2009), and the maximum duration of migration of Kirtland's warblers ( *Setophaga kirtlandii* ; distance of about 2200 km) was 13–23 days (Ewert et al. 2012). Comparable data is not available for yellow warblers, but observation of yellow warblers on their wintering grounds in Mexico well into the second week of May (Valdez‐Juarez et al. 2019) suggests that their spring migration takes a similar amount of time.

In birds, the timing of arrival on the breeding grounds can be influenced by various factors. In this study, we found limited evidence that the timing of arrival on the breeding grounds varies with growing degree days (GDD), which is closely linked to the temperature on the breeding grounds and the stage of vegetation phenology at the time of arrival (Hakkinen et al. 1998; Linkosalo 1999). Female arrival dates did not vary across the 3 years of this study, despite marked differences in GDD among years. While our findings are consistent with some studies that concluded that climate conditions on breeding grounds are not a good predictor of arrival phenology (e.g., Both et al. 2006; Weidinger and Král 2007; Møller et al. 2008). Other studies argued that breeding ground temperature can be an important factor in predicting the arrival of migratory songbirds on the breeding grounds (e.g., Ahola et al. 2004; Marra et al. 2005; Mazerolle et al. 2011; Mihoub et al. 2012; Connare and Islam 2022). Previous work with yellow warblers demonstrated that wind conditions experienced during migration affect the arrival timing of males (Drake et al. 2014). While arrival dates for yellow warbler males are easily recorded due to their more vocal and territorial nature, arrival data for females are much more difficult to obtain. In this study, we attempted to carefully document female arrival. By analyzing 3 years of detailed female arrival data, we found limited evidence that wind experienced during migration significantly influenced the arrival of females, even though conditions on migration seemed likely to influence the timing of arrival of both males and females. However, in these 3 years, there was little variation in the speed of crosswinds on the Pacific flyway during the time of yellow warbler spring migration, which could explain the lack of support for the effect of wind on female arrival timing in this study.

Upon arrival on the breeding grounds, female songbirds rapidly transition from migration to reproductive physiology in order to lay eggs (Williams 2012b). Songbirds are often considered “income breeders” (e.g., Smith and Moore 2003; Langin et al. 2006) that fuel this transition, egg production, and parental care using energy acquired on the breeding grounds. However, recently, Pavlik et al. (2021) have demonstrated that some females can arrive on the breeding grounds in a relatively advanced stage of yolk precursor production, suggesting they use stored energy (Drent and Daan 1980) to partially finance the early stages of reproduction. Here, we confirm that the reproductive state (plasma triglyceride concentration) on arrival influences the time interval between arrival and egg laying. Variation was associated with both plasma triglyceride levels and crosswinds faced by females during the 14‐day period before arriving on the breeding grounds (Figure 2). However, crosswind effects on the interval between arrival and egg laying were not caused by changes in reproductive state attributable to crosswinds experienced during migration. Plasma triglyceride levels in female yellow warblers were not correlated with crosswinds experienced during the 14‐day period before arrival on the breeding grounds. Moreover, we did not find a correlation between GDD on arrival and either reproductive state (plasma triglyceride) on arrival or the interval between arrival and egg laying.

Carryover effects from crosswinds experienced during migration on the timing of breeding and reproductive success of yellow warblers are likely to arise due to their effect on arrival date and how rapidly birds can transition from a migratory to a reproductive physiological state. The interval between arrival on the breeding territory and laying of the first egg can fluctuate based on multiple factors. Later arriving females could compensate for their late arrival by shortening this interval, perhaps by taking advantage of phenologically advanced vegetation, higher temperatures, and more abundant food (e.g., Meijer et al. 1999; Salvante et al. 2007; Visser et al. 2009; Descamps et al. 2011; Dunn et al. 2011; Nightingale et al. 2023). Similarly, females arriving in better breeding conditions (body condition and/or more advanced reproductive state) could take advantage of this asset by being able to shorten this interval (e.g., Drent and Daan 1980; Rowe et al. 1994; Descamps et al. 2011; Hennin et al. 2016; Lamarre et al. 2017). The ability of some, but not all, females to arrive on the breeding grounds in a more advanced reproductive state (Pavlik et al. 2021) further complicates the relationship between arrival time and first egg date. Arriving in more advanced stages of yolk precursor production could be modulated by the condition or quality of individual females and/or through differences in the speed of migration and time spent at stopover sites among individuals. Females who arrive with elevated triglyceride concentrations are perhaps able to observe environmental cues during the final stages of migration and adjust the speed of migration to allow for diverting more energy toward changes in reproductive physiology, especially during unfavorable weather events (e.g., strong headwind). However, neither residual mass nor plasma triglyceride concentration appears to be directly influenced by the wind conditions on migration. While mass may be a poor measure of an individual's condition (Green 2001; Labocha and Hayes 2012; Beauchamp et al. 2021), obtaining information for a wider range of plasma metabolites could help better explain any potential differences in physiological state that may be associated with variation in wind conditions on migration. Similarly, if precise spatial and temporal data on flight and stopover events during migration could be obtained, our ability to parse energetic dynamics could be greatly increased.

In this study, we demonstrate that crosswinds experienced during the last 14 days before arrival on the breeding grounds influence the interval between arrival and the start of egg laying. How wind conditions may affect the ability of females to shorten the interval between arrival and egg laying remains unclear because crosswind effects were independent of when females arrived and their residual mass or reproductive state on arrival. Similarly, it remains to be uncovered what the mechanisms are that allow some females to arrive with high plasma triglyceride levels. Nevertheless, this study highlights the importance of the carryover effects of migration on the timing of reproduction in determining the productivity of a migratory songbird. We provide further evidence that the benefits associated with initiating reproduction early can lead to selective pressures that favor individuals capable of a fast transition from migratory to reproductive physiology and of minimizing the delay between arrival and egg laying.

## Author Contributions

**Michal Pavlik:** conceptualization (equal), data curation (lead), formal analysis (lead), investigation (equal), methodology (equal), visualization (lead), writing – original draft (lead), writing – review and editing (equal). **Tony D. Williams:** conceptualization (equal), data curation (supporting), formal analysis (supporting), investigation (equal), methodology (equal), writing – original draft (supporting), writing – review and editing (equal). **David J. Green:** conceptualization (equal), data curation (supporting), formal analysis (equal), investigation (equal), methodology (equal), writing – original draft (supporting), writing – review and editing (equal).

## Conflicts of Interest

The authors declare no conflicts of interest.

## Supporting information


Figures S1–S5

## Data Availability

Data will be deposited in the Dryad Digital Repository.
