# Supplementary material for: Crosswinds During Spring Migration Carryover and Influence the Time Interval Between Arrival and Laying in a Neotropical Migrant
Source: Ecol Evol. 2025 May 8;15(5):e71230. doi: 10.1002/ece3.71230 (PMC12059623; doi:10.1002/ece3.71230)
Supplement: Supplementary file 1 — Figures S1–S5 [file ECE3-15-e71230-s001.docx]

Supplementary material


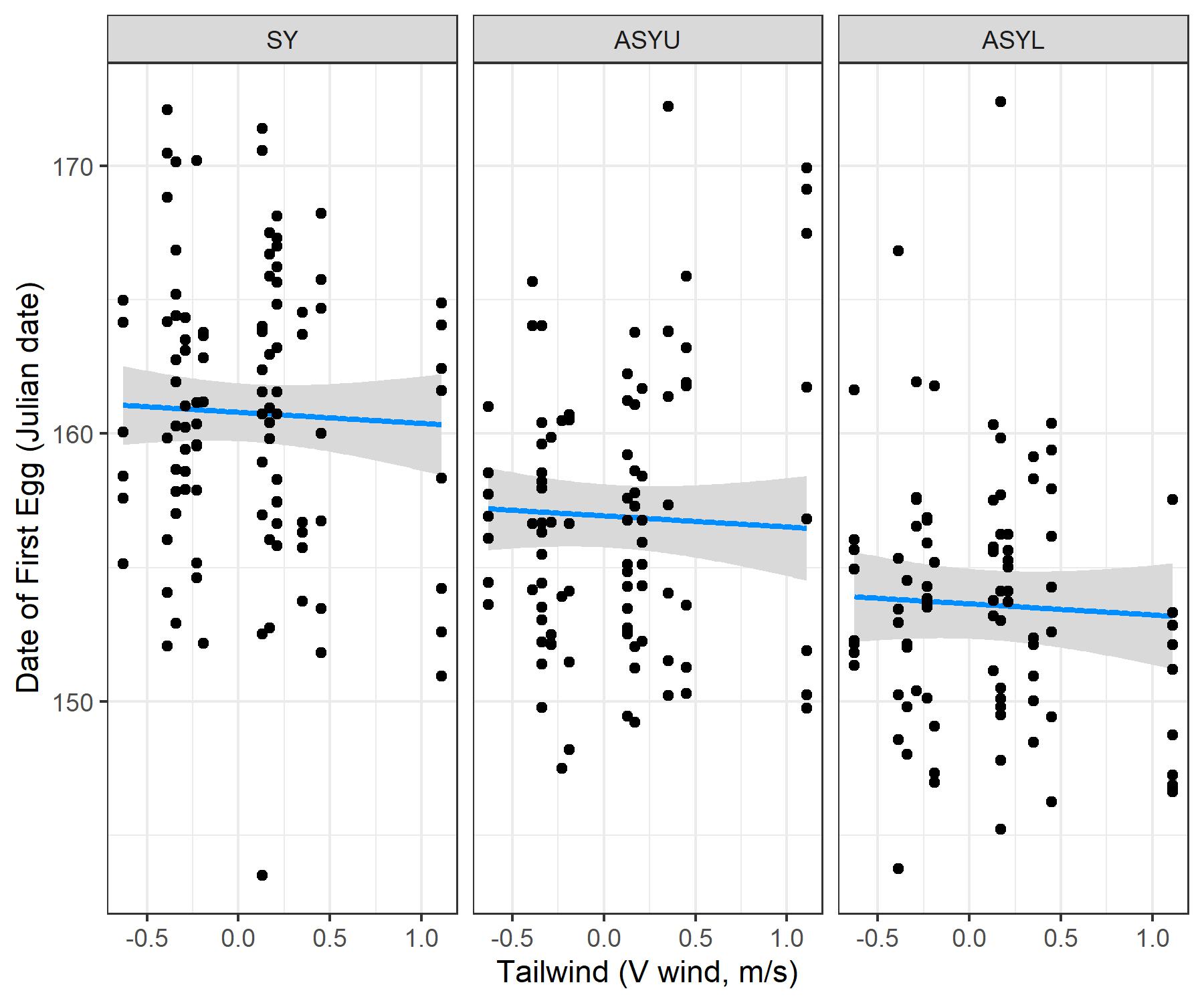


Figure S1: Relationship between southerly tailwind (V-wind) speed on the migration flyway during the 14-day period May 18-31 and date of first egg for yellow warblers breeding in Revelstoke, British Columbia, from 2005-2017. SY females are second year females breeding for the first time, ASYU are after second year females of at least two years of age that are new to the study area, and ASYL are local after second year females that are returning to the study area. We present the model predictions (line), 95% confidence interval (shading) and partial residuals (points) from the top model in the candidate set that included age class, standardized average tailwind (V-wind) and crosswind (U-wind) speed as fixed effects and individual ID and Year as random terms.


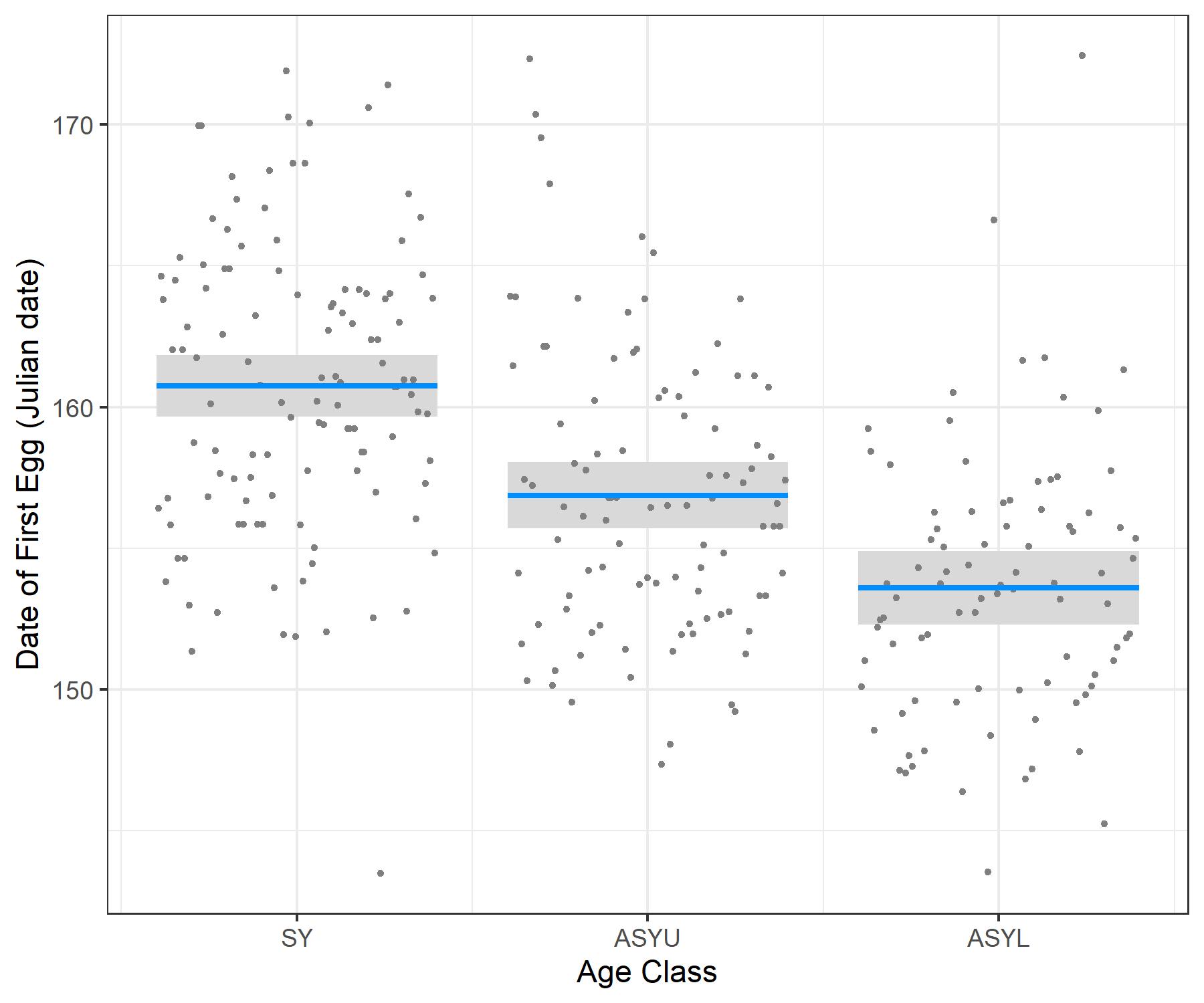


Figure S2: Age class effects on the date of the first egg for yellow warblers breeding in Revelstoke, British Columbia, from 2005-2017. SY females are second year females breeding for the first time, ASYU are after second year females of at least two years of age that are new to the study area, and ASYL are local after second year females that are returning to the study area. We present the model predictions (line), 95% confidence interval (shading) and partial residuals (points) from the top model in the candidate set that included age class, standardized average tailwind (V-wind) and crosswind (U-wind) speed as fixed effects and individual ID and Year as random terms.


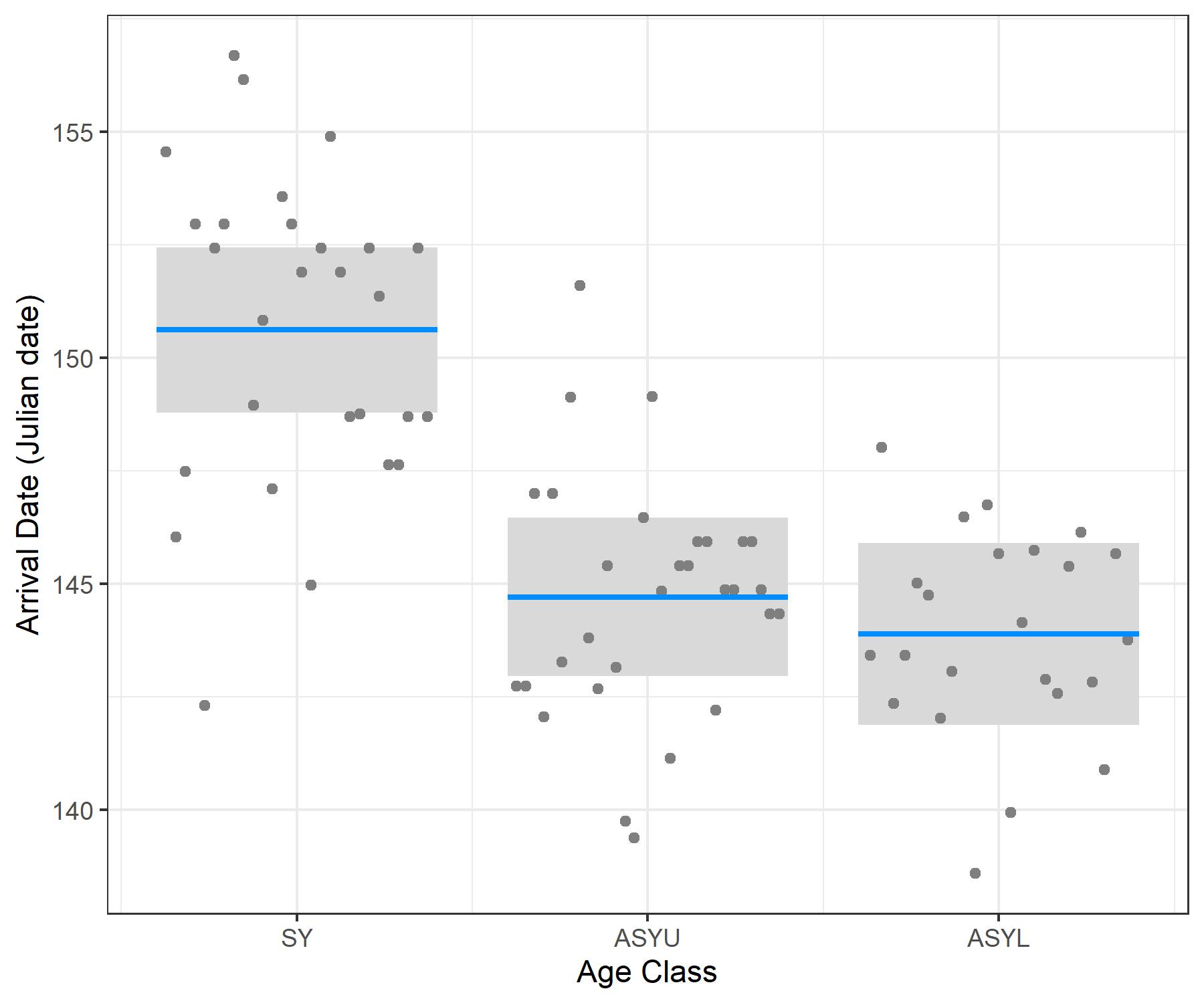


Figure S3: Age class effects on the arrival date of yellow warblers breeding in Revelstoke, British Columbia, from 2015-2017. SY females are second year females breeding for the first time, ASYU are after second year females of at least two years of age that are new to the study area, and ASYL are local after second year females that are returning to the study area. We present the model predictions (line), 95% confidence interval (shading) and partial residuals (points) from the top model in the candidate set that included age class as a fixed effect and individual ID as a random term.


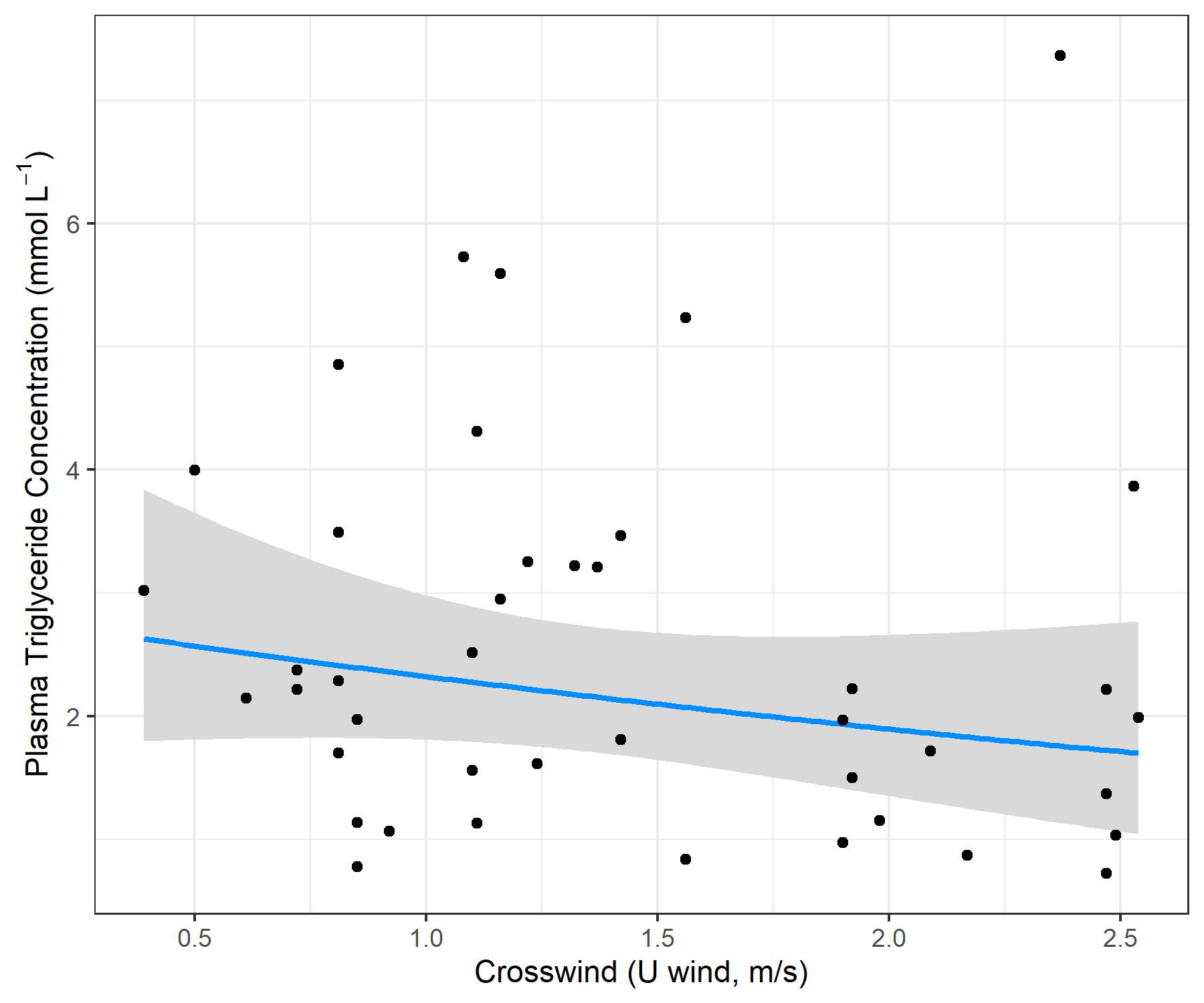


Figure S4: Relationship between crosswind (U-wind) speed on the entire flyway in the two weeks prior to the arrival of individuals and the plasma triglyceride concentration of female yellow warblers sampled within 2 days of arrival on the breeding grounds (n = 42). We present the model predictions (line), 95% confidence interval (shading) and partial residuals (points) from the top model in the candidate set that included the standardized arrival date, standardized average crosswind speed (Uwind_ind_) and timing of capture terms (days after arrival, DAA).


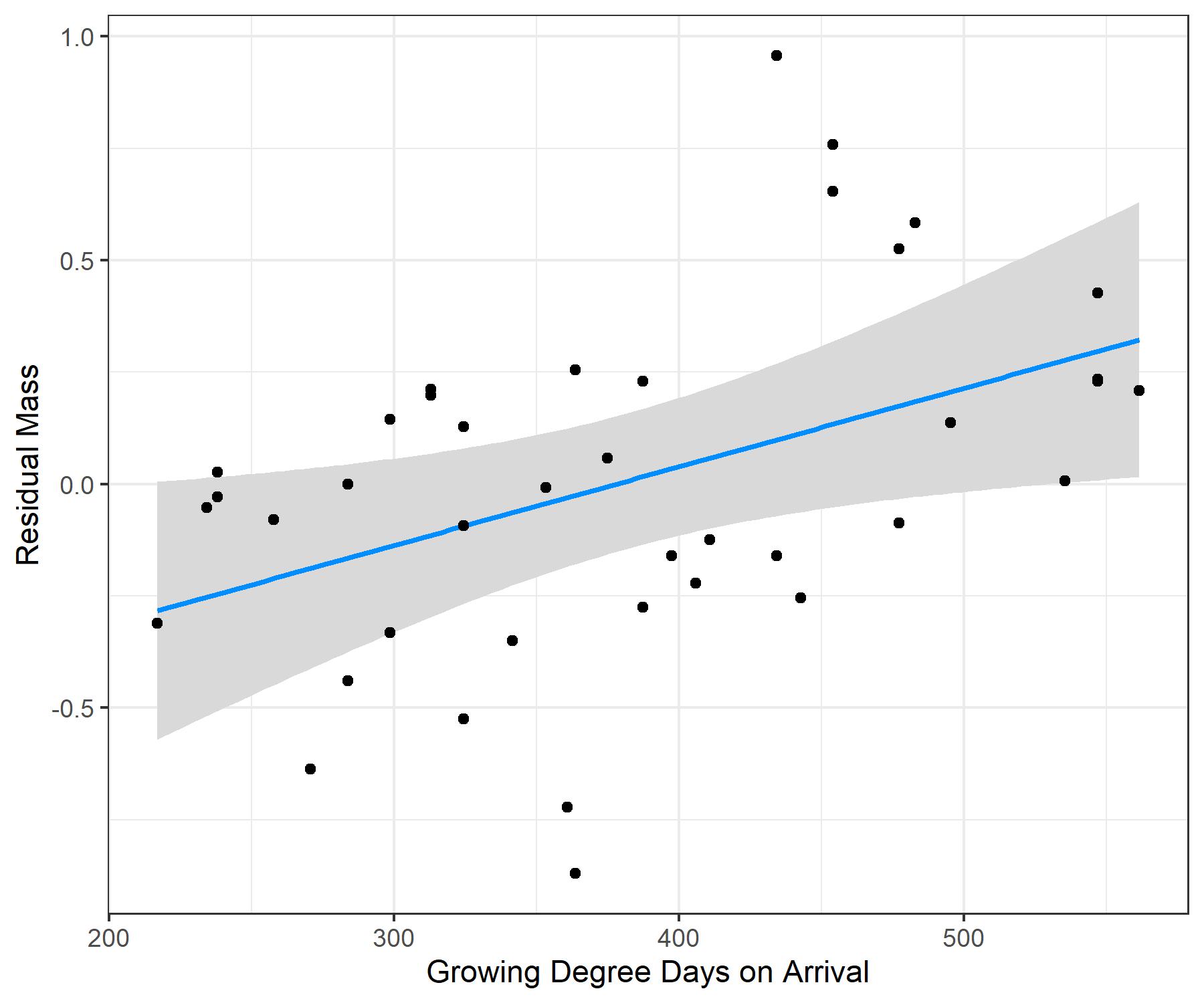


Figure S5: Relationship between cumulative growing degree days on the day of arrival on the breeding grounds and the mass of female yellow warblers sampled within 2 days of arrival on the breeding grounds (n = 40). Mass estimates are residuals from a mass tarsus-regression. We present the model predictions (line), 95% confidence interval (shading) and partial residuals (points) from the top model in the candidate set that included the standardized growing degrees on arrival (GDD_ind_) as fixed effects and individual ID as a random term.
